# Retraction: Plasma D-Dimer Levels Are Associated with Stroke Subtypes and Infarction Volume in Patients with Acute Ischemic Stroke

**DOI:** 10.1371/journal.pone.0266428

**Published:** 2022-03-30

**Authors:** The PLOS ONE Editors

Following the publication of this article [1], similarities were noted between this article and manuscripts submitted by other research groups, including [2–5].

When these issues were raised with the authors, the first author requested that the article [1] be retracted. They stated that a third-party company provided assistance with language translation, editing and submission of this article. Additionally, they stated that the raw data are no longer available.

The similarities between [1] and work by other researchers call into question the validity and provenance of the reported results and the adherence of this article to the PLOS Authorship policy. In light of these issues, the *PLOS ONE* Editors retract this article [1].

WJZ and JS agreed with the retraction and apologized for the issues with the published article.
